# Assessment of a 44 Gene Classifier for the Evaluation of Chronic Fatigue Syndrome from Peripheral Blood Mononuclear Cell Gene Expression

**DOI:** 10.1371/journal.pone.0016872

**Published:** 2011-03-30

**Authors:** Daniel Frampton, Jonathan Kerr, Tim J. Harrison, Paul Kellam

**Affiliations:** 1 Department of Infection, Division of Infection and Immunity, University College London, London, United Kingdom; 2 Division of Clinical Sciences, St George's University of London, London, United Kingdom; 3 Department of Internal Medicine, University College London Medical School, London, United Kingdom; 4 The Wellcome Trust Genome Campus, The Wellcome Trust Sanger Institute, Cambridge, United Kingdom; Memorial Sloan Kettering Cancer Center, United States of America

## Abstract

Chronic fatigue syndrome (CFS) is a clinically defined illness estimated to affect millions of people worldwide causing significant morbidity and an annual cost of billions of dollars. Currently there are no laboratory-based diagnostic methods for CFS. However, differences in gene expression profiles between CFS patients and healthy persons have been reported in the literature. Using mRNA relative quantities for 44 previously identified reporter genes taken from a large dataset comprising both CFS patients and healthy volunteers, we derived a gene profile scoring metric to accurately classify CFS and healthy samples. This metric out-performed any of the reporter genes used individually as a classifier of CFS.

To determine whether the reporter genes were robust across populations, we applied this metric to classify a separate blind dataset of mRNA relative quantities from a new population of CFS patients and healthy persons with limited success. Although the metric was able to successfully classify roughly two-thirds of both CFS and healthy samples correctly, the level of misclassification was high. We conclude many of the previously identified reporter genes are study-specific and thus cannot be used as a broad CFS diagnostic.

## Introduction

Chronic fatigue syndrome (CFS) is a clinically defined illness with a broad range of symptoms including severe and debilitating fatigue, muscle pain, sleep disruption, difficulties with concentration, memory impairment and headaches. It is estimated to affect 0.4% of the population in Europe and North America [1] and cost $9 billion annually in lost productivity in the USA alone [2]. The cause and pathogenesis of CFS remain poorly understood, although various infectious triggers have been proposed.

There are currently no specific laboratory-based tests that provide a robust diagnosis of CFS. However, previous studies indicate significant differences in the patterns of gene expression in peripheral blood leukocytes between patients with CFS and healthy individuals [3]–[10]. Although many of these studies have not detailed predictive sets of genes that could be used to make a diagnosis of CFS on the basis of their expression, we showed previously that the expression levels of 88 “CFS reporter genes”, identified using microarrays, could assign individuals to CFS disease or healthy control groups following quantitative PCR of PBMC RNA [7], [8].

Microarray analysis has been used frequently to identify groups of genes associated with various diseases, including infectious diseases [11], autoimmune diseases and cancer [12]. In such studies, a microarray dataset featuring tens of thousands of genes is computationally reduced to several hundred genes found to be significantly differentially expressed between healthy and diseased individuals, or between different stages of disease. Computational methods, such as support vector machines (e.g. [13]), artificial neural networks (e.g. [14]) and simple selective naïve Bayes classifiers [15], are able to identify such gene sets for disease classification. These methods require training the underlying statistical models on data representative of diseased and healthy phenotypes in order to make such predictions. Ideally, models are trained on a well-characterised dataset and evaluated using a separate, preferably blinded, test set consisting of new samples from diseased and healthy individuals. The use of different non-array based methods for quantification of gene expression, such as reverse transcription polymerase chain reaction (RT-PCR) is also desirable. On this basis, a gene profile can be formally assessed as a multiplex diagnostic tool.

Here, we have undertaken such an analysis to determine the predictive power of our ‘CFS signature genes’ identified previously [7], [8]. We have assessed the CFS disease predictive genes in the original study data and in a new blinded sample set of CFS disease and healthy control samples. We show, using a variety of methods, that these genes do not identify robustly patients with CFS disease.

## Results

### CFS class prediction using a 44 gene classifier

To develop a robust CFS diagnostic metric, we used as a training set the mRNA relative quantities (RQ, defined as 2^−ΔΔCT^) for the 44 most discriminating reporter genes identified previously by us [7], [8] (Table S1). The data were normalised to GAPDH and to a calibrator sample. Initially, we assessed the ability of each of the individual reporter genes to be used as an accurate predictor of CFS disease in this dataset of PBMC gene expression, determined for 56 patients suffering from CFS and 75 healthy volunteers.

**Figure 1:**
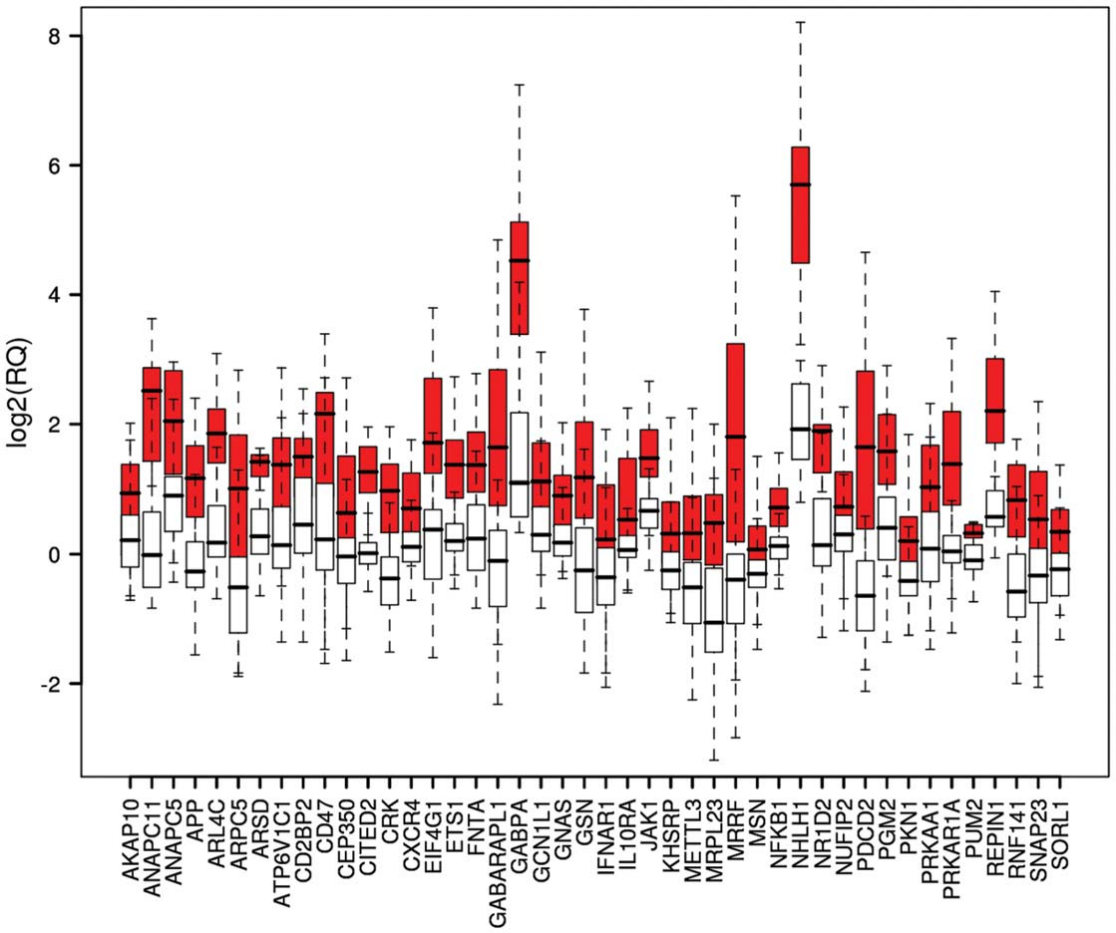
A boxplot illustrating the distribution of log_2_(RQ) values for each of the reporter genes in the training set. Distributions for CFS samples are shown in red, healthy samples in white. The boxes represent the inter-quartile range (25–75%) of the data with the median being shown as a solid horizontal line within each box. Whiskers extend to 1.5 times the inter-quartile range. The data is log_2_ scaled and outliers omitted for purposes of clarity.

For each of the 44 reporter genes, we assessed the distribution of RQ values, finding that each of the genes was expressed at higher levels in the CFS samples than controls (Figure 1). In some cases, the RQ values of the reporter genes were clearly separate and the mean RQ differed significantly between CFS and controls, for example ANAPC11. For other classifier genes, although the mean RQ values differed significantly between CFS and control samples, the separation of the individual RQ values was not so distinct, for example MRRF.

For each of the genes we performed receiver operator curve (ROC) analysis, using different RQ value cut-offs to produce ROC curves and a corresponding assessment of sensitivity, specificity, true and false positive rates (TPR, FPR) for each gene. We used the results of the ROC analyses to identify an RQ value cut off for each gene that maximised the true positive, and minimised the false positive, classification of CFS samples. A sample was classified as CFS-positive if its RQ value was greater than the calculated cut-off.

**Figure 2:**
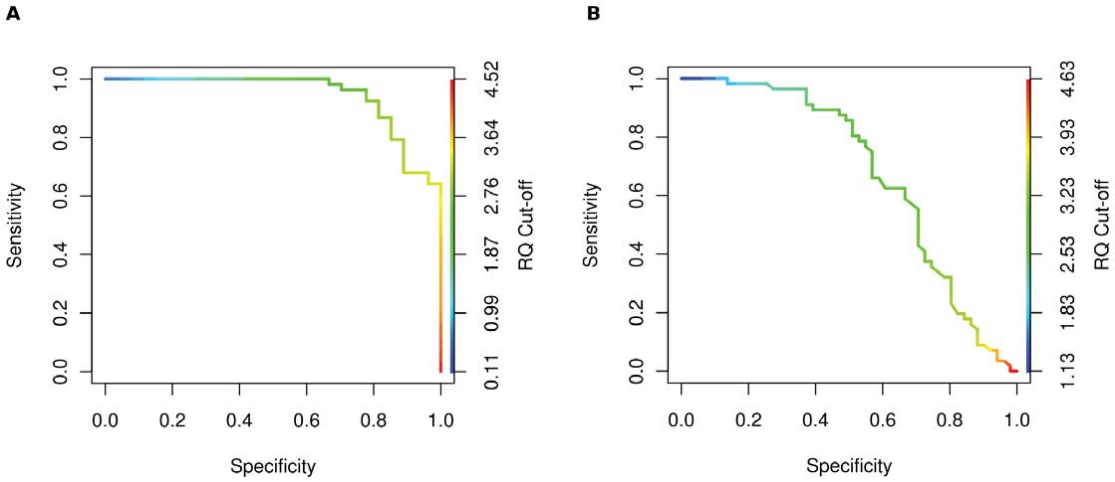
ROC curves obtained when using (A) ARL4C and (B) NUFIP2 as CFS predictors on the training set. The graphs are coloured according to the scale bar on the right hand y-axes to indicate the RQ value cut-offs associated with each pair of sensitivity and specificity values at that particular point in the ROC curve. The AUCs were found to be: (A) 0.94 and (B) 0.67.

**Figure 3:**
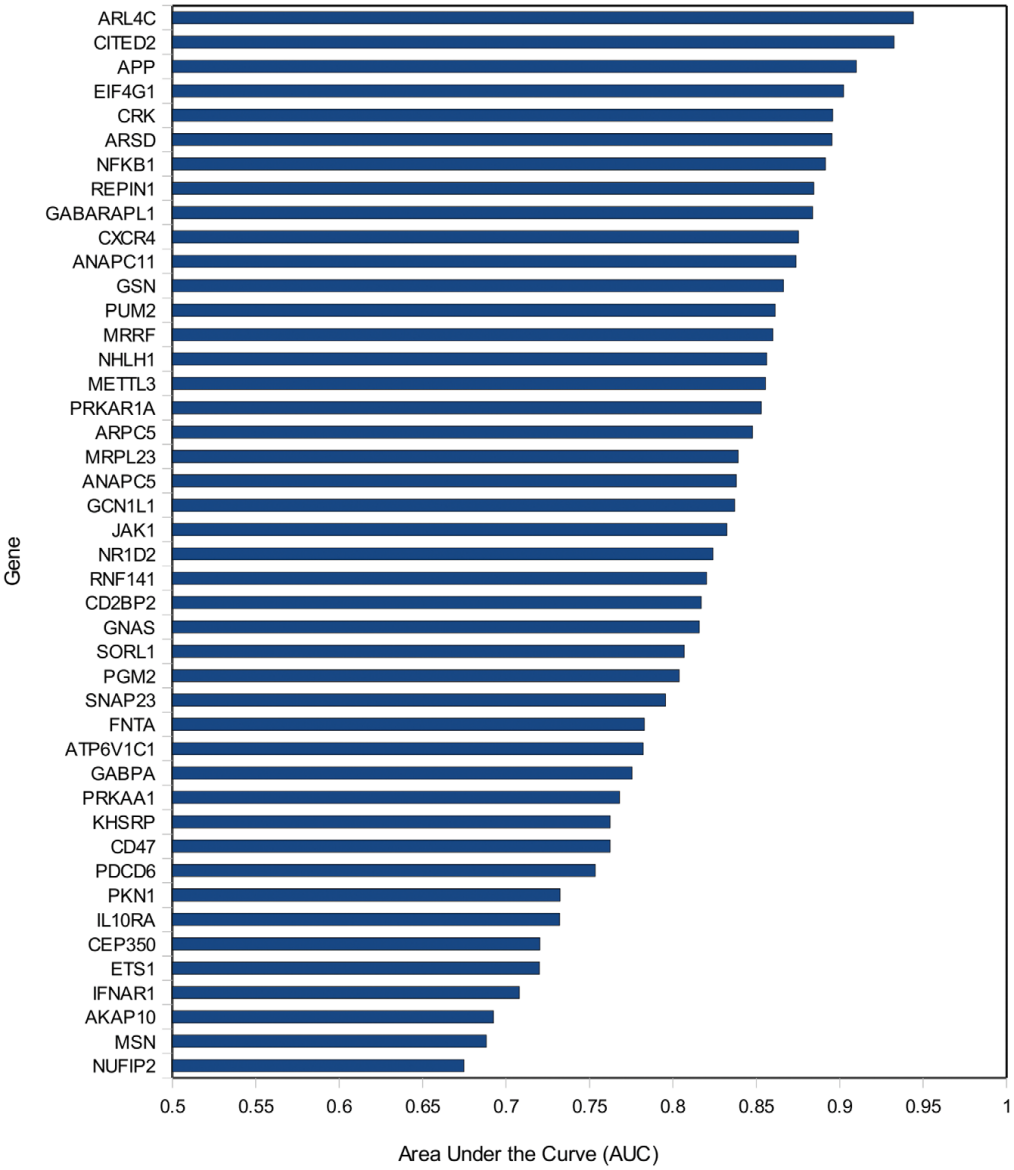
Area under the curve (AUC) values for each of the reporter genes when used as CFS predictors on the training set. All perform better than random (AUC = 0.5).

Consistent with the distribution of RQ values for each gene (Figure 1) the area under the curve (AUC) from the ROC analysis for the 44 reporter genes varies substantially, from 0.67 to 0.94 (Figures 2, 3 and Table S2). ARL4C was found to be the best CFS predictor (AUC = 0.94), whilst NUFIP2 performs poorly (AUC = 0.67); thus, NUFIP2 performs only marginally better than a random predictor (AUC = 0.5). Consequently, the individual 44 reporter genes vary considerably in their ability to predict CFS in the training set accurately, although all perform better than a random predictor (Figure 3). Those with high AUCs yield fewer false positives at low RQ cut-offs and fewer false negatives at higher RQ cut-offs.

**Figure 4:**
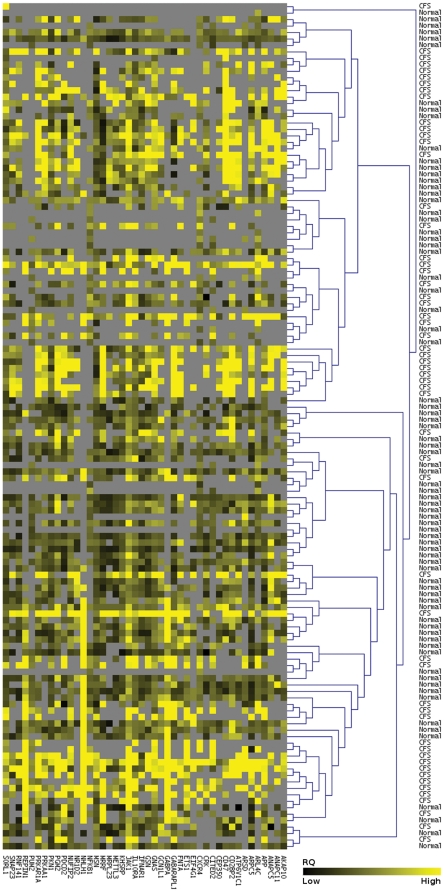
Hierarchical clustering across samples of RQ values from the training set by Euclidean distance with average linkage. Reporter genes are arranged in columns, samples in rows. Missing values are shown in grey.

Using individual genes as classifiers is often problematic due to missing data, where gene expression levels are not available for all patients and individual variation of single gene expression confounds predictive power. Thus, in principle, no single reporter gene would be able to classify CFS accurately across all samples. We therefore explored the data structure of the 44 gene expression set using unsupervised hierarchically clustering of the PCR-derived RQ gene expression levels in the training set using Euclidean distance (Figure 4). Although there is clearly an underlying structure to the data, with many of the CFS and control samples clustering in distinct groups, there is also clearly overlap between the two groups. This is most likely due to missing data and occasionally large RQ outliers skewing the clustering. Consistent with this observation, the 44 reporter genes could not produce a CFS classifier using support vector machines by training on a subset of the training set and assessing the predictive power on a separate subset (data not shown). To minimise the effect of missing gene expression levels in each sample, we filtered the training set to include only samples for which there were at least 22 different RQ gene expression values (i.e. 50% of the reporter genes). However, this did not improve the ability to predict CFS cases using hierarchical or k-means clustering, or SVMs.

### Gene Profile Score Metric

Because standard clustering and classification methods were unable to classify the training set, we developed a scoring metric based around a gene-profiling approach. For each sample, a gene profile was generated using a binary classification of CFS or control for each reporter gene: a sample with a RQ value greater than the defined gene specific cut-off corresponding to a 5% false positive rate determined from the earlier ROC analysis was assigned a ‘1’ (“present”), or otherwise a ‘0’ (“absent”). Any missing data values were assigned a ‘0’. The per gene score is summed over the 44 genes, resulting in a profile score of between 0 and 44 for each sample. Samples with high scores should be over represented for CFS disease whilst samples with low scores all should be healthy controls.

**Figure 5:**
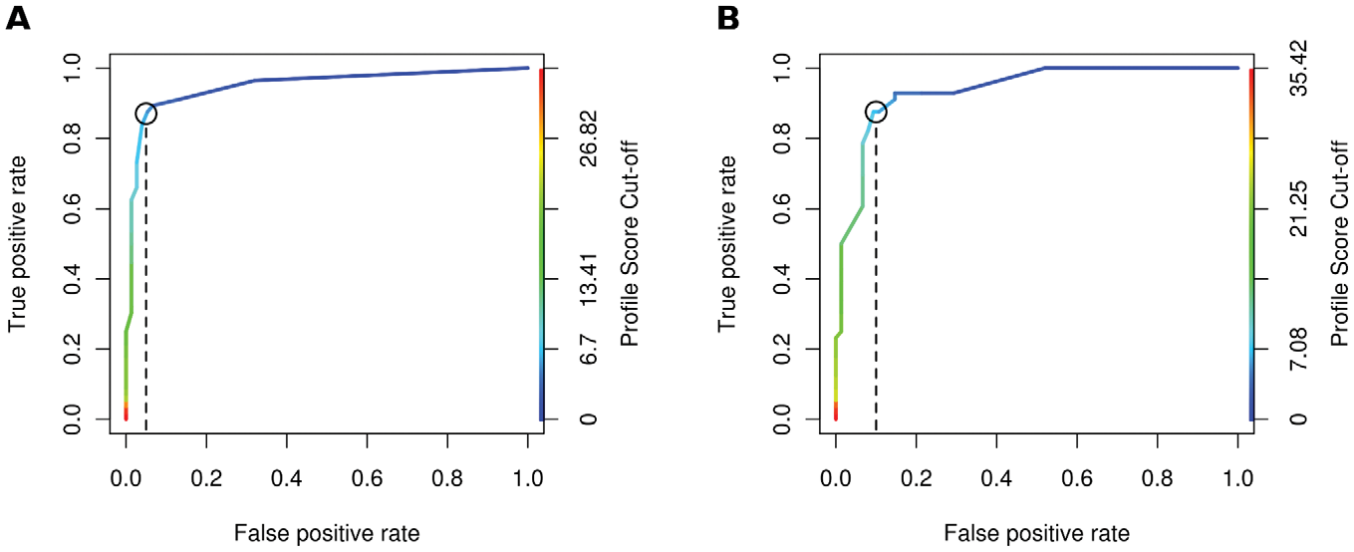
ROC curves for the gene profile score metric using a false positive rate (FPR) on the training set of (A) 5% and (B) 10%. For more detail, see Materials and Methods. The graph is coloured to indicate the gene profile score cut-offs associated with each pair of true and false positive rates at that particular point in the ROC curve. The range of profile cut-off scores are coloured on the right hand y-axes of each graph. The profile score cut-offs used to classify the test set were derived by selecting scores yielding FPR's of (A) 5% and (B) 10%, as indicated by the dashed lines.

We assessed the ability of this metric to differentiate between CFS and healthy samples in the training set again using ROC analysis on the profile score, obtaining true and false positive rates for a range of profile score cut-offs (Figure 5). This method results in an AUC of 0.95, with 47 CFS (84%) and 72 healthy controls (96%) correctly predicted in the 131 sample training set. This therefore represents a better predictor of CFS than any of the genes used individually (Figure 3).

We applied this gene profile score metric to analyse a blind study dataset, selecting a profile score cut-off of 5, above which samples were classed as CFS disease and equal to or below were classed as healthy controls. At this level of score threshold, the training set gave a FPR of 5%. Under these criteria, our predictor assigned from the 128 blinded samples, 32 CFS positive samples, of which 22 were true-positives (69%) and 10 were false-positives (31%) (Table 1). Correspondingly, it predicted 96 healthy samples of which 51 were true negatives (53%) and 45 were false negatives (47%). Overall, this resulted in 73 (58%) correct classifications (TP+TN), based on our gene expression scoring metric. An empirical assessment of the blinded test set based on expert assessment of the RQ values resulted in the correct class prediction of 79 of 128 samples (61%).

**Table 1:** The performance of the gene profile score metric in classifying samples in the test set.

Method		Positive	Negative
**5% FPR**	**True text**	22	51
	**False text**	10	45
**10% FPR**	**True text**	43	37
	**False text**	24	24

Cut-offs were used on the test set corresponding to false positive rates (FPR) of 5% and 10% on the training set.

**Figure 6:**
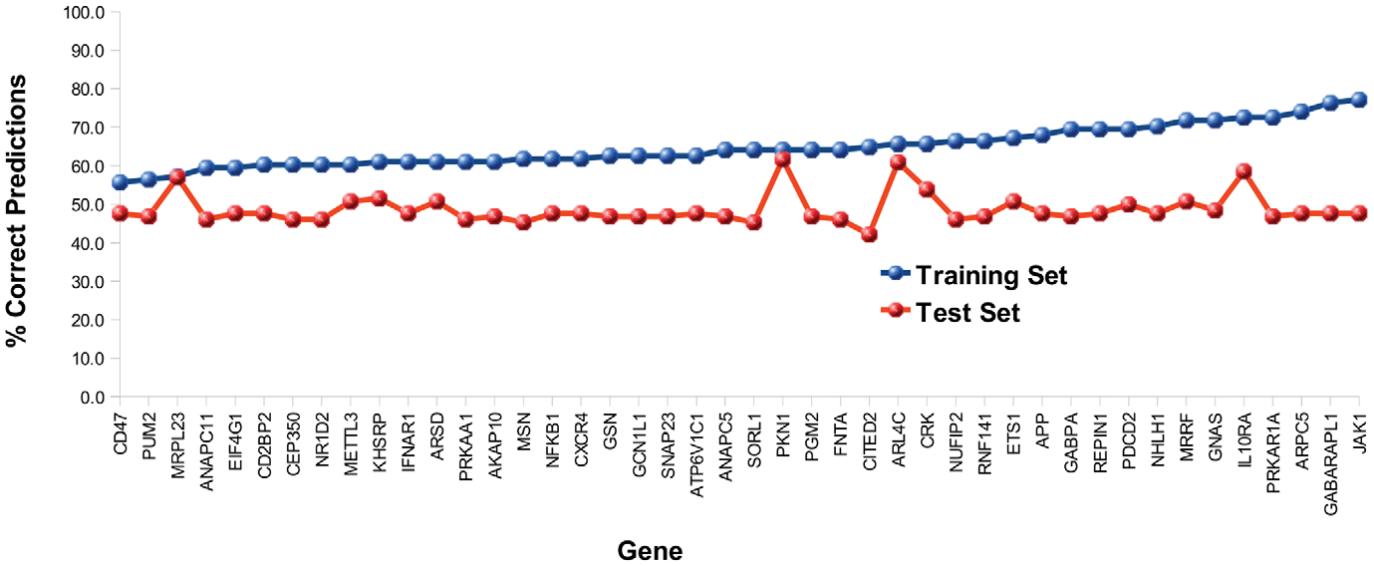
Percentage of predictions made correctly by each of the reporter genes using cut-offs corresponding to a FPR of 5% on the training set (blue) and the test set (red).

To determine if the predictive power that was evident in the training data was lost on the test data due to a subset of poorly performing genes we looked at the number of correctly predicted samples per gene (Figure 6). This showed that except for 3 genes (MRPL23, PKN1 and ARL4C) all other genes had significantly lower predictive power in the test set. Although a number of reporter genes are good CFS predictors in the training set, the majority perform no better than random, 32 of the 44 making more incorrect than correct predictions on the test set (Tables S3, S4). Those that are good CFS predictors in the training set are not necessarily good predictors in the test set: there is no correlation between the number of correct predictions made by each reporter gene individually across the two data sets (Pearson's correlation coefficient = 0.07).

To determine if the calibrator reference sample confounded the RQ-based analysis we investigated the properties of normalized real time ΔCT values. Performing ROC analysis and generating profile scores for the ΔCT values, we found no significant difference in predictive outcome for the 44 reporter genes in terms of their ability to classify CFS and healthy samples, either when used as individual classifiers or by combining them to generate profile scores (Figure S1, Tables S5, S6).

For the training set, mean RQ values are significantly higher in CFS than healthy samples for each of the 44 reporter genes (Welch 2-sample t-test, P<0.01). However, no significant difference is observed between the means for any of these genes in the test set (Welch 2-sample t-test, all P>0.01). Thus, although the reporter genes could be used (either by profile score or, to a lesser extent, individually) to classify CFS in the original study, they are not robust in a new sample population.

## Discussion

Here we have assessed the ability of a proposed 44 gene classifier [8] to discriminate between CFS patients and healthy control individuals. This classifier was able to discriminate correctly between CFS and healthy control samples in 95% of the training samples. However, when assessed on a new, blinded 128 sample test set only 58% of samples were predicted correctly. Importantly, a high number of false-positive predictions were made, with 31% of CFS-positive predictions being from healthy volunteers. In addition, a high number of false negative predictions were made, 57% of the CFS samples being predicted as healthy controls. Therefore, with the methods used here we cannot predict CFS disease based on the analysis of expression of 44 classifier genes in the peripheral blood.

There may be several reasons for the poor performance on the blinded test set for what, as far as the training set is concerned, would otherwise be considered a good predictive metric. Firstly, the low 5% FPR per gene in the development of cut-off scores for the scoring metric may have resulted in over-fitting to the training set. This seems unlikely, however, as similar results were observed based on a more relaxed training set FPR of 10% with the training set still producing a classifying AUC of 0.94 (Figure 5). Although roughly two-thirds (43/67, 64%) of the CFS samples from the blinded test set were classified correctly using a profile score cut-off corresponding to a FPR of 10%, the rate of misclassification was higher (24/67, 36%; Table 1). Secondly, there may be fundamental biological differences between the training and test set due to for example, population stratification, age, onset of CFS or other factors which confound both clustering and gene-profiling approaches. In the worst case, the 44 reporter genes may not be representative beyond the small study in which they were identified [7].

The determination of microarray study specific gene classifiers has lead to the proposed identification of various CFS reporter genes, based on microarray analysis of gene expression [3]–[10]. There is little overlap in the gene sets, a reflection of the difference between studies. Larger CFS microarray studies, together with analysis by comparison with different control groups, may help in identify CFS disease-classifying gene sets. Alternatively, a meta-analysis of existing CFS data sets would provide a valuable extension with the potential to identify gene expression signatures for formal assessment as disease classifiers in new samples, as outlined here.

## Materials and Methods

### Datasets

Two datasets of quantitative real time RT-PCR values pre-processed as mRNA relative quantities (RQ), defined as 2^−ΔΔCT^ for 44 reporter genes were used in this study. The first dataset, was generated from PBMC samples taken from 57 CFS patients and 75 healthy volunteers and had previously been reported [8]. This was used as a training set. The second study was from a blinded study and was used as a test set. This comprised PBMC samples from 64 CFS patients and 64 healthy volunteers. This sample set was blinded and the disease classification was not assessed until the classes were predicted computationally. The data for the blinded study was collected via clinical questionnaires as outlined previously [8]. The patients were provided with paper copies for completion at home and subsequent return by post to the clinical centre. Data from the questionnaires was collated and used for clinical characterisation. The diagnosis of CFS was based on the Fukuda criteria and this diagnosis was made by CFS clinical experts. The blinded study was approved by the Wandsworth Research Ethics Committee, St George's Hospital, London, SW17 0QT. Verbal consent was given by all subjects for their information to be stored and used for this study.

### Analysis

Hierarchical and k-means clustering of RQ values was performed using the TIGR MeV software suite [16]. Support vector machines (SVM) were also created using MeV, using the training set to train the SVM and the test set to evaluate the accuracy of SVM classification.

Receiver operator curve (ROC) analysis was performed using the ROCR library [17] within R/BioConductor [18]. Individual reporter genes were used as CFS predictors for the training set, the corresponding sensitivity, specificity, true and false positive rates were determined for all possible RQ cut-offs (from the smallest to largest RQ value). Areas under the curve were determined for each of the reporter genes as a measure of prediction accuracy. The same procedure was used to evaluate the gene profile score metric, outlined below.

### Gene profile score metric

A gene profile score metric was generated as follows. Firstly, RQ cut-offs were determined for each reporter gene such that the associated false-positive rate of CFS prediction in the training set, using that gene alone, was 5%. These cut-offs were used to create binary profiles (44 digits in length) for each sample, with a ‘1’ indicating that particular sample had an RQ greater than the cut-off and a 0, otherwise (either a lower RQ or a missing value).

The norm of the profile was then calculated by summing all 44 scores to give the “gene profile score”. Thus, if the reporter genes are accurate predictors of CFS, samples from CFS patients would yield a higher profile score than those from healthy volunteers. The gene profile score metric itself was evaluated using ROC analysis using the training set. From this analysis. a profile score cut-off was generated at which level of the metric yielded a 5% false positive rate when classifying the training set. Profile scores were then generated for the blinded test set and samples were classified as “CFS” or “healthy” according to this cut-off.

We assessed the effect of altering the RQ cut-off score such that the false positive rate for both individual reporter genes and for the resultant gene profile score metric was 10% in terms of classifying CFS and healthy samples in the training set.

## Supporting Information

Figure S1 ROC curves for CFS classification at a 5% FPR of the training set by gene profile score using ΔCT (red) and RQ (blue) values. Both yield AUCs of 0.95.
(TIFF)

Table S1 The 44 CFS reporter genes used in this study.
(DOC)

Table S2 Area under the curve (AUC) values for each of the reporter genes when used as CFS predictors on the training set.
(DOC)

Table S3 Performance of each of the reporter genes used individually as CFS predictors on the training set.
(DOC)

Table S4 Performance of each of the reporter genes used individually as CFS predictors on the test set.
(DOC)

Table S5 Relative performance of each of the scoring metrics.
(DOC)

Table S6 AUCs for the individual reporter genes used as predictors on the training and test sets, using RQ and ΔCT values.
(DOC)
