# Supplementary figures and images for: Assessment of a 44 Gene Classifier for the Evaluation of Chronic Fatigue Syndrome from Peripheral Blood Mononuclear Cell Gene Expression
Source: PLoS One. 2011 Mar 30;6(3):e16872. doi: 10.1371/journal.pone.0016872 (PMC3068152; doi:10.1371/journal.pone.0016872)

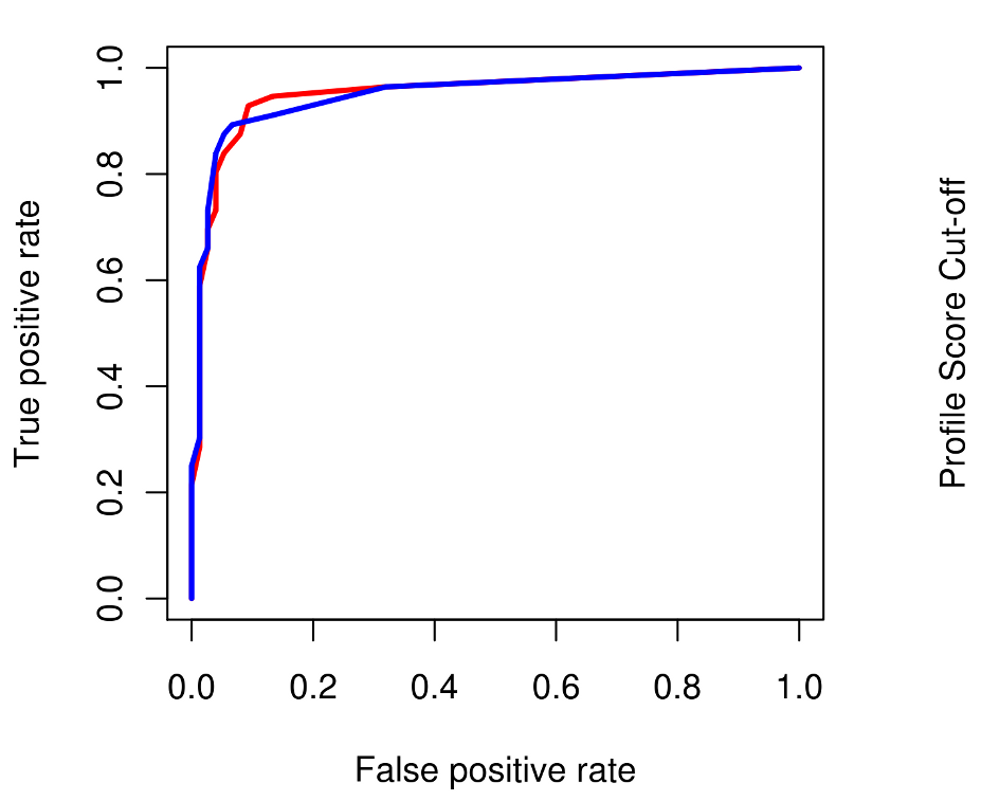

Supplement: Figure S1 — ROC curves for CFS classification at a 5% FPR of the training set by gene profile score using ΔCT (red) and RQ (blue) values. Both yield AUCs of 0.95. (TIFF) [file pone.0016872.s001.tif]
