# Supplementary material for: Assessment of a 44 Gene Classifier for the Evaluation of Chronic Fatigue Syndrome from Peripheral Blood Mononuclear Cell Gene Expression
Source: PLoS One. 2011 Mar 30;6(3):e16872. doi: 10.1371/journal.pone.0016872 (PMC3068152; doi:10.1371/journal.pone.0016872)
Supplement: Table S1 — The 44 CFS reporter genes used in this study. (DOC) [file pone.0016872.s002.doc]

**Table S1.**  The 44 CFS reporter genes used in this study.
