# Supplementary material for: Assessment of a 44 Gene Classifier for the Evaluation of Chronic Fatigue Syndrome from Peripheral Blood Mononuclear Cell Gene Expression
Source: PLoS One. 2011 Mar 30;6(3):e16872. doi: 10.1371/journal.pone.0016872 (PMC3068152; doi:10.1371/journal.pone.0016872)
Supplement: Table S2 — Area under the curve (AUC) values for each of the reporter genes when used as CFS predictors on the training set. (DOC) [file pone.0016872.s003.doc]

**Table S2.**  Area under the curve (AUC) values for each of the reporter genes when used as CFS predictors on the training set.
