# Supplementary material for: Assessment of a 44 Gene Classifier for the Evaluation of Chronic Fatigue Syndrome from Peripheral Blood Mononuclear Cell Gene Expression
Source: PLoS One. 2011 Mar 30;6(3):e16872. doi: 10.1371/journal.pone.0016872 (PMC3068152; doi:10.1371/journal.pone.0016872)
Supplement: Table S4 — Performance of each of the reporter genes used individually as CFS predictors on the test set. (DOC) [file pone.0016872.s005.doc]

**Table S4.** Performance of each of the reporter genes used individually as CFS predictors on the test set.

TP = true positives, TN = true negatives, FP = false positives, FN = false negatives. “% Correct” refers to the percentage of predictions made correctly. Cut-offs were determined for each gene by setting the FPR to 5% in the training set.
