# Supplementary material for: Assessment of a 44 Gene Classifier for the Evaluation of Chronic Fatigue Syndrome from Peripheral Blood Mononuclear Cell Gene Expression
Source: PLoS One. 2011 Mar 30;6(3):e16872. doi: 10.1371/journal.pone.0016872 (PMC3068152; doi:10.1371/journal.pone.0016872)
Supplement: Table S5 — Relative performance of each of the scoring metrics. (DOC) [file pone.0016872.s006.doc]

**Table S5.**  Relative performance of each of the scoring metrics.

TP: true positives; FP: false positives; TN: true negatives; FN: false negatives. ∆CT values were mean-normalised by reporter gene before applying cut-offs to improve cross comparison between datasets.
