# Supplementary material for: Assessment of a 44 Gene Classifier for the Evaluation of Chronic Fatigue Syndrome from Peripheral Blood Mononuclear Cell Gene Expression
Source: PLoS One. 2011 Mar 30;6(3):e16872. doi: 10.1371/journal.pone.0016872 (PMC3068152; doi:10.1371/journal.pone.0016872)
Supplement: Table S6 — AUCs for the individual reporter genes used as predictors on the training and test sets, using RQ and ΔCT values. (DOC) [file pone.0016872.s007.doc]

**Table S6.** AUCs for the individual reporter genes used as predictors on the training and test sets, using RQ and ∆CT values.

ROC analysis was performed on the blind test set after annotation (i.e. “unblind”). There is little difference in performance between using either RQ or ∆CT values for either dataset.
